# Response-adapted treatment with upfront high-dose chemotherapy followed by autologous stem-cell transplantation rescue or consolidation phase high-dose methotrexate for primary central nervous system lymphoma: a long-term mono-center study

**DOI:** 10.1186/s40064-016-1954-6

**Published:** 2016-03-10

**Authors:** Yoko Nakasu, Koichi Mitsuya, Nakamasa Hayashi, Ikue Okamura, Keita Mori, Terukazu Enami, Raine Tatara, Satoshi Nakasu, Takashi Ikeda

**Affiliations:** Division of Neurosurgery, Shizuoka Cancer Center, Nagaizumi, Sunto, Shizuoka, 4118777 Japan; Division of Stem Cell Transplantation, Shizuoka Cancer Center, Nagaizumi, Sunto, Shizuoka, 4118777 Japan; Division of Biostatics, Shizuoka Cancer Center, Nagaizumi, Sunto, Shizuoka, 4118777 Japan; Division of Neurooncology, Kusatsu General Hospital, Yabase, Kusatsu, Shiga, 5250066 Japan

**Keywords:** Autologous stem-cell transplant, Chemotherapy, Methotrexate, Primary central nervous system lymphoma, Radiotherapy, Rituximab

## Abstract

Treatment regimens for primary central nervous system lymphoma (PCNSL) include high-dose methotrexate (HD-MTX)-based chemotherapy, with or without radiotherapy and are based on studies of selected patient groups. This retrospective study assessed a consistent strategy of response-adapted protocol applied for patients including age >65 years in a cancer center for 10 years longitudinally. Case notes were studied of 61 consecutively treated patients with PCNSL histologically diagnosed between 2003 and 2013. Clinical follow-up during and after treatment included neurologic examination and magnetic resonance imaging. Of the patients studied, 14.8 % (9/61) were clinically unfit for chemotherapy; the remaining 85.2 % (52/61) of patients were treated with HD-MTX. Of these patients, 58 % (30/52) achieved an initial complete response, with a median survival of 100.1 months. Of these response-adapted patients, 33 % (10/30) were <65 years and were treated with upfront high-dose chemotherapy and autologous stem-cell transplantation (HDC-ASCT). The remaining response-adapted patients included 53 % (16/30) who were ≥65 years underwent consolidation with HD-MTX, and 14 % (4/30) who chose radiotherapy. The median survival of patients with HDC-ASCT had not yet been reached compared with 67.6 months for patients with HD-MTX consolidation treatment (p = 0.26). At the end of the study, 75 % (39/52) of patients had died mainly owing to progression or relapse of PCNSL. Multivariate analysis showed that age younger than 65 years (p = 0.02) and complete response for up-front HD-MTX (p = 0.001) were independent prognostic indicators of overall survival. In conclusion, this single-center retrospective clinical study has shown that treatment of PCNSL with upfront HDC-ASCT and consolidation phase HD-MTX monotherapy may be feasible, even for elderly patients in a routine clinical setting, using the three-step selection by eligibility and response to initial HD-MTX, and age threshold of 65 years for ASCT.

## Background

Primary central nervous system lymphoma (PCNSL) is a rare form of extra-nodal lymphoma that accounts for approximately 0.8–6.6 % of primary intracranial neoplasms (Deckert and Paulus 2007). Ninety-five percent of PCNSL are diffuse large B-cell lymphoma (DLBCL) (Deckert and Paulus 2007). PCNSL affects all ages, with a peak incidence, in immunocompetent subjects, during the sixth and seventh decades of life (Deckert and Paulus 2007). Histologically, PCNSL may be low-grade or high-grade, with the histological grade reflecting biological aggressiveness. The optimal treatment strategy for newly diagnosed PCNSL remains controversial, and long-term, disability-free survival remains poor (Weller et al. 2012; Morris et al. 2013; Wen 2012; Ponzoni et al. 2014). The poor prognosis in PCNSL is particularly found in patients over the age of 60 years, who comprise the majority of cases.

Current treatment regimens for PCNSL include high-dose methotrexate (HD-MTX)-based chemotherapy with or without radiotherapy. Previously, whole brain radiation therapy (WBRT) had played the most important role achieving median survival time between 11 and 18 months (Shibamoto et al. 2014; Nelson et al. 1992). The addition of chemotherapy with HD-MTX has resulted in patient survival beyond 30 months (Glass et al. 1996; O’Brien et al. 2006). However, both HD-MTX and WBRT, individually and in combination, are associated with acute and long-term neurotoxicity and cognitive dysfunction, which are more common in patients who are older than 60 years (Abrey et al. 1998).

Although it is acknowledged that HD-MTX-based chemotherapy improves patient survival in PCNSL, the optimal chemotherapy regimen, the role of WBRT and feasibility of high-dose chemotherapy with autologous stem-cell transplantation (HDC-ASCT) rescue are less well defined (Hoang-Xuan et al. 2015). The evidence for patient treatment regimens is based on studies in highly selected groups of patients. However, many patients do not meet the selection criteria of clinical trials, as they are often elderly with poor performance status, impaired cognitive function and rapid neurological progression. In clinical practice, prompt treatment decisions are required to prevent irreversible deterioration. Therefore, elderly patients have been hardly included in randomized clinical trials. Poor patient survival in PCNSL has many causes, including ineligibility for systemic therapy, biological resistance to initial therapies, acute and long-term adverse events, and frequent relapses that may be refractory to second-line treatments. There are few studies on response-adapted treatment with upfront HDC-ASCT rescue or consolidation HD-MTX for patients of all ages with PCNSL in routine clinical setting, and length of follow-up after primary treatment has yet to be defined.

In our clinical center, a three-stage intent-to-treat strategy for patients of all ages with PCNSL has been used consistently for more than 10 years. This three-stage response-adapted treatment strategy includes upfront HD-MTX followed by HDC-ASCT rescue or consolidation phase HD-MTX treatment. This retrospective clinical study was undertaken to determine whether this response-adapted treatment strategy is feasible and whether it contributes to survival of patients with PCNSL including elderly in routine clinical setting.

## Methods

### Patients

We examined the records of 61 consecutive patients with primary central nervous system lymphoma (PCNSL), diagnosed between June 2003 and May 2013 at our institution. Patient data were collected in June 2015. The Institute Review Board approved the study protocol and patient treatment protocols.

All patients included in this study had a histological diagnosis of PCNSL. All patients had a staging evaluation before treatment. Investigations included gadolinium-enhanced magnetic resonance imaging (MRI) of the brain and spinal cord and computed tomography (CT) scans of the chest, abdomen and pelvis, creatinine clearance, and human immunodeficiency virus (HIV) testing. Patients underwent lumbar puncture, ultrasonic examination of the testes, bone marrow biopsy, and fluorodeoxyglucose-positron emission tomography (FDG-PET) imaging. A slit-lamp ophthalmologic examination was carried out routinely.

### Treatment strategy (Fig. 1)

The following three-stage patient selection process was used:

**Fig. 1:**
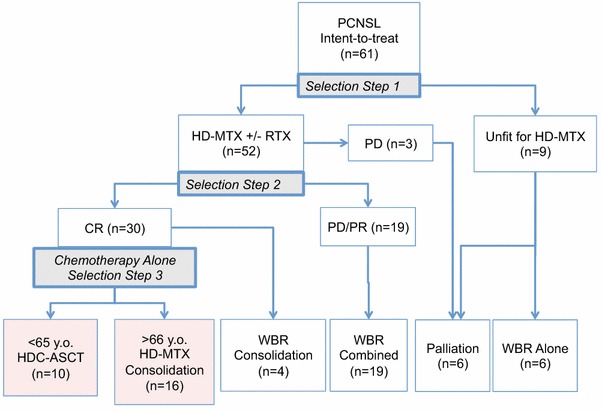
Treatment schema and distribution of patients. *AE* adverse events, *CR* complete response, *HD-MTX* high-dose methotrexate, *HDC* high-dose chemotherapy, *ASCT* autologous stem-cell transplantation, *PD* progressive disease, *PR* partial response, *RTX* rituximab, *WBR* whole brain radiotherapy

- Selection Step 1: Eligibility for initial HD-MTX. Throughout the study period, all patients received treatment with HD-MTX as their initial treatment, if they were eligible for treatment. If a patient was considered unfit to tolerate HD-MTX on the basis of poor performance status and/or systemic complications, the patient underwent WBRT or palliative therapy.
- Selection Step 2: Response to the initial HD-MTX. We set response-adapted treatments. For patients who achieved a complete response (CR) from the initial HD-MTX, the intention was that they should proceed to consolidation chemotherapy deferring WBRT. For patients who had a partial response (PR) or progressive disease (PD), WBRT was recommended.
- Selection Step 3: Consolidation regimen by patient age. Patients with intention-to-treat using chemotherapy-alone were subdivided to HDC-ASCT rescue for patients under 65 years of age and HD-MTX consolidation therapy for patients who were 65 years and older.

### HD-MTX monotherapy and response-adapted therapy

HD-MTX (at a dose of 3.5 g/m^2^ intravenously over 6 h on day 1) with calcium folinate rescue was repeated every 2 weeks for a maximum of six cycles. Patients received corticosteroid (betamethasone) at a dose of 2 mg/day in day 1 of each course, and in day 1 of following courses if clinically indicated.

The HD-MTX dose was adjusted according to the patient’s creatinine clearance measurements. MTX was injected into CSF through an Ommaya reservoir intra-ventricular catheter system or via lumbar puncture once a week between cycles of HD-MTX. If the brain biopsy specimen showed CD20-positive B-lymphocytes, patients received rituximab 375 mg/m^2^ for a maximum of six doses between the weeks of HD-MTX therapy.

Patients who had a CR with HD-MTX were assigned to one of two chemotherapy-alone treatments according to their age (Selection Step 3). Patients under 65 years of age without systemic disease underwent HDC-ASCT; patients over 65 years of age were treated with HD-MTX at a dose of 1 g/m^2^ once every 2 months on four occasions (Fig. 1). If a patient had PD or PR after the second or fourth course of HD-MTX, the patient proceeded to WBRT at 40 to 50 Gy.

### Upfront HDC-ASCT

G-CSF was used for mobilization of peripheral blood progenitor cells after the hyper-CVAD/MA protocol during bone marrow recovery. High-dose chemotherapy before ASCT included one of three regimens: ICE (ifomide, carboplatin, etoposide), MCEC (ranimustine, carboplatin, etoposide, cyclophosphamide), or MEAM (ranimustine, cytarabine, etoposide, melphalan) (Wilson et al. 1992; Imai et al. 2005; Abrey et al. 2003; Takasaki et al. 2013).

### Patient follow-up

All patients were followed-up clinically after treatment with a neurologic examination and gadolinium-enhanced MRI. Patients were examined at least once a month using MRI during initial HD-MTX therapy. Patients were evaluated every 2 months during the first 2 years, and then every 3 months for a year, and every 4–6 months for at least years, for a total of 10 years after initial therapy. Treatment of recurrent PCNSL utilized re-challenge with HD-MTX monotherapy, administration of carboplatin and etoposide, administration of alkylating chemotherapy, or WBRT for chemo-resistant patients.

Response to treatment was recorded as follows: CR was defined as the absence of enhanced lesions or bright lesions of diffusion-weighted MR images and no ocular involvement by lymphoma; PR was defined as a 50 % or more decrease in size of lesions on MR images; PD was defined as neurological deterioration or 25 % or more increase in size of lesions or any new lesions on imaging. Patients with recurrent disease following CR underwent thorough diagnostic and staging evaluation again, and received chemotherapy, WBRT, or palliative therapy according to their neurologic and systemic conditions and previous history of treatment.

### Statistical analysis

The date of diagnosis was taken as the date of the diagnostic biopsy or surgical tumor excision. The date of any relapse after CR was recorded along with details of further treatment and the date and cause of death. For evaluation of delayed neurotoxicity, the global functionality of patients was quantified with Karnofsky performance score (KPS). Cox proportional hazard model was applied to assess the strength of association as for overall survival (OS). The analyzed variables included patient age, gender, eligibility for initial HD-MTX, CR by initial HD-MTX treatment, the Memorial Sloan Kettering Cancer Center (MSKCC) prognosis score, and the International Extranodal Lymphoma Study Group (IELSG) prognosis score. The OS was defined as the time from diagnosis to death from any cause. Progression-free survival (PFS) was defined as the time from diagnosis to first documentation of disease progression on imaging or death from lymphoma. Survival times were calculated using the Kaplan–Meier method, and survival curves were compared using the log-rank test. The level of significance (p) was 0.05. Results were analyzed using commercially available statistical software (JMP v.8, SAS Institute Inc., Tokyo, Japan).

## Results

### Patient background and outcome

**Fig. 2:**
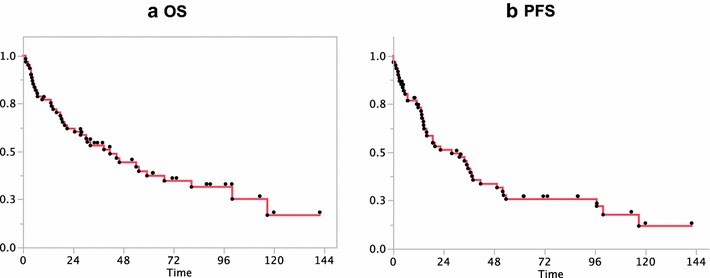
Kaplan–Meier curves of all patients. **a** Kaplan–Meier curve shows the median overall survival (OS) of all patients is 41.6 months (95 % CI 21.1–67.6 months). **b** Kaplan–Meier curve shows the median progression-free survival (PFS) of 27.9 months (95 % CI 14.7–38 months)

Between June 2003 and May 2013, 61 patients (38 men and 23 women) were newly diagnosed with PCNSL (Table 1). The median age at diagnosis was 69 years (range 42–80 years), only three patients (4.9 %) were under 50 years of age. Fifty-eight tumors were high-grade, diffuse large B-cell lymphoma (DLBCL), and three were diagnosed as high-grade lymphoma with no specific phenotype. All patients had parenchymal brain involvement by primary lymphoma. Concomitant ocular involvement was detected by slit-lamp examination in two patients. All patients were immunocompetent and negative for HIV serology. The median OS estimated for the entire study population was 41.6 months (95 % CI 21.1–67.6 months), and the median PFS was 27.9 months (95 % CI 14.7–38 months) (Fig. 2a, b). When grouped by age, patients between 50 and 59 years had a median survival of 116.5 months (95 % CI 21.1 months, upper limit unavailable at the time of observation). Patients 70 years or older had a shorter median OS of 14.4 months (95 % CI 5.4–30.2 months) (log rank p < 0.0001).

**Table 1:** Characteristics of the patients according to accomplished first-line treatment

Characteristics	All	Elidible to initial HD-MTX			Non-eligible
		HDC-ASCT	HD-MTX consolidation	HD-MTX + WBR	WBR alone or palliation
Total number	61	10	16	23	9
Gender (man:woman)	38:23	8:2	9:7	14:9	5:4
Age (years) median (range)	69 (42–80)	57 (46–62)	72 (55–80)	66 (42–79)	74 (70–79)
KPS (%) median (range)	60 (30–100)	60 (40–90)	60 (40–90)	50 (30–100)	30 (30–40)
MSKCC					
Class I	3	2	0	1	0
Class II	20	4	8	7	0
Class III	38	4	8	15	9
IELSG score					
0 & 1	11	3	4	4	0
2 & 3	32	6	10	12	1
4 & 5	18	1	2	7	1
Response to HD-MTX					
CR	30	10	16	4	–
PR	7	0	0	7	–
PD	12	0	0	12	–
WBR number	29	0	0	23	6
Median dose (range)	50 (30–59.4)	–	–	50 (40–59.4)	40 (30–50)

*KPS* Karnofsky performance status, *MSKCC* Memorial Sloan Kettering Cancer Center, *IELSG* International Extranodal Lymphoma Study Group, *HD-MTX* high-dose methotrexate, *HDC-ASCT* high-dose chemotherapy with autologous stem cell transplant, *WBR* whole brain radiotherapy, *CR* complete response, *PR* partial response, *PD* progressive disease

### First-line treatments

Fifty-two of 61 patients (85.2 %) were eligible for and received initial HD-MTX chemotherapy (Selection Step 1). Forty patients received HD-MTX in combination with rituximab (Fig. 1). Fifty patients received intrathecal injection of MTX. Of the 52 patients treated with HD-MTX, 41 patients (78.8 %) completed an initial three to six cycles of HD-MTX. Thirty (73.2 %) of 41 patients achieved CR, and 26 of these patients were given further consolidation treatment with chemotherapy alone, deferring WBRT (Selection Step 2). Ten patients proceeded to HDC-ASCT, and 16 patients had HD-MTX consolidation therapy according to their age (Selection Step 3). Four of 30 patients with CR chose WBRT for their consolidation therapy.

All patients with CR rapidly improved in their levels of consciousness and focal neurological deficits during the first course of HD-MTX. After completion of the initial treatment, two patients with CR had mild residual neurologic signs, including one patient with a dystonic posture of the left fingers, and another patient with mild hemiparesis. Seven (17.1 %) of 41 patients achieved PR, and they underwent WBRT. Fifteen (28.8 %) of 52 patients failed to respond to the initial HD-MTX therapy; 12 patients with PD immediately proceeded to WBRT, and three patients received palliative care. Nine of 61 patients (14.8 %) were considered unfit to undergo HD-MTX therapy at Selection Step 1 because of poor performance status, severe cardiac compromise, advanced renal failure or hepatic failure. Six of these patients received WBRT, and three received palliative care.

**Fig. 3:**
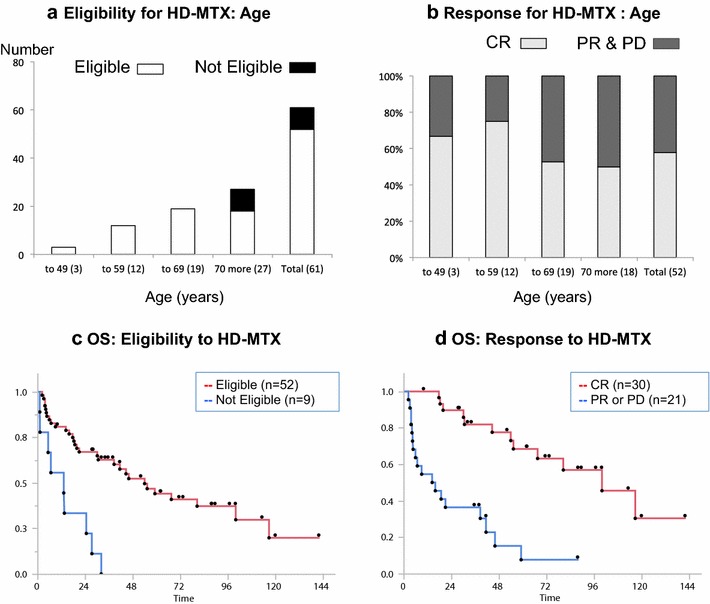
Eligibility and responses for HD-MTX. **a** Age distribution of patients and eligibility for HD-MTX. For initial HD-MTX therapy, 85.2 % patients were eligible (*white*), and nine were unfit for treatment (black). **b** In total, 57.7 % patients achieved a complete response (*light gray*), and 22 other responses (*dark gray*) for initial HD-MTX. **c** Kaplan–Meier estimate of overall survival by eligibility for initial HD-MTX. Median survival for patients eligible for treatment was 54.1 months (95 % CI 30.7–100.1 months) compared with 13.3 months (95 % CI 1.2–27.5 months) for those not eligible (Log rank p < 0.0001). **d** Kaplan–Meier estimate of overall survival by the response to the initial HT-MTX. Median survival for patients with complete response was 100.1 months (95 % CI 55.4 months to upper limit not available), compared with those with other responses 15.2 months (95 % CI 4.5–41.6 months (Log rank p < 0.0001). *HD-MTX* high-dose methotrexate, *CR* complete response, *PR* partial response, *PD* progressive disease, *OS* overall survival

Figure 3a shows the number of patients and their eligibility for initial HD-MTX therapy by age group. The ineligible patients were all over 70 years of age. The percentage of patients with CR following initial HD-MTX decreased in the elderly patient group. However, more than 50 % of the patients in the seventh and eighth decades of life responded well to treatment (Fig. 3b). Fifty-two patients, who were eligible for initial HD-MTX, showed an increased median OS of 54.1 (95 % CI 30.7–100.1 months) compared with nine patients who were ineligible for HD-MTX, with a median OS of 13.3 months (95 % CI 1.2–27.5 months) (log-rank p < 0.0001) (Fig. 3c). Patients with CR had a median OS of 100.1 months (95 % CI 55.4 months, upper limit is not available), which was longer than the OS of patients with PR or PD (log-rank p < 0.0001) (Fig. 3d).

**Fig. 4:**
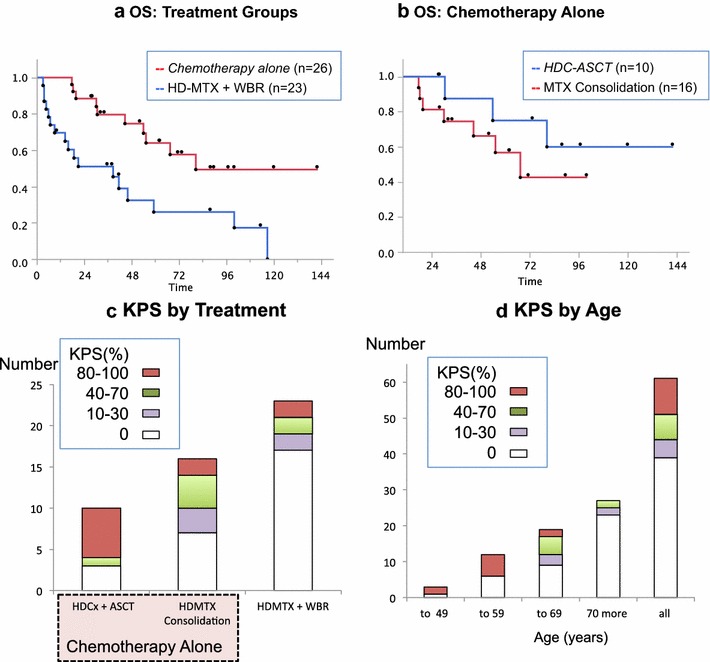
Kaplan–Meier estimate of overall survival according to initial treatment groups. **a** Patients treated with chemotherapy alone had a median overall survival 80.6 months (95 % CI 54.1 months to upper limit not available) compared with patients received HD-MTX + WBR 38.7 months (95 % CI 9.1–59.3 months) (Log rank p = 0.0037). **b** Of 26 patients treated with chemotherapy alone, ten patients underwent HDC-ASCT and 16 received HD-MTX consolidation. Patients treated with HDC-ASCT showed a survival curve that did not reach the median; patients treated with HD-MTX consolidation had a median overall survival 67.6 months (95 % CI 30.2 months to upper limit not available) (Log rank p = 0.2618). **c** Karnofsky Performance Score of the patients at the last follow-up. More than half of the patients treated with HDC-ASCT presented with 80–100 % KPS (*red*). **d** Distribution of KPS by age groups. Twenty-two patients were alive longer than 2 years, and 10 (45.5 %) of 22 patients had returned to their normal lives with a KPS of 80–100 %. *WBR* whole brain radiotherapy, *OS* overall survival, *KPS* Karnofsky performance status, *HD-MTX* high-dose methotrexate, *HDC-ASCT* high-dose chemotherapy with autologous stem-cell transplant, *MTX* methotrexate

When sub-divided according to treatment group, 26 patients treated with chemotherapy alone showed an increase in median OS to 80.6 months (95 % CI 54.1 months, upper limit is not available) compared with patients who received HD-MTX plus WBRT (log-rank p = 0.0037) (Fig. 4a). Kaplan-Meier survival curves of ten patients with upfront HDC-ASCT had not yet reached to its median value, and 16 with HD-MTX consolidation therapy had a median OS of 67.6 months (95 % CI 30.2 months, upper limit is not available) (log-rank p = 0.2648) (Fig. 4b). Cox-proportional hazard model showed that age younger than 65 years (p = 0.02) and complete response for up-front HD-MTX (p = 0.001) were the independent factors of overall survival (Table 2).

**Table 2:** Multivariate analysis of parameters associated with overall survival

Parameters	OS		p
	HR	95 % CI	
Gender			
Women versus men	1.321	0.652–2.622	0.432
Age			
Over 65 versus under 65	2.721	1.157–6.671	0.0216*
HD-MTX eligibility			
Eligible versus non-eligible	0.873	0.359–2.205	0.768
Response to HD-MTX			
CR versus others	0.140	0.053–0.343	0.001*
MSKCC class			
1 & 2 versus 3	0.504	0.202–1.186	0.118
IELSG score			
0 to 3 versus 4 & 5	1.13	0.501–2.555	0.766

*CI* confidence interval, *HR* hazard ratio

* Statistically significant value

### Early toxicity of first-line chemotherapy

Of the 52 patients who received HD-MTX therapy, only one patient experienced Common Terminology for Adverse Events Adverse Event (CTCAE) grade-4 heart failure, and he died of lymphoma progression within 2 months following the start of HD-MTX. Otherwise, most patients occasionally showed CTCAE grade-1 or -2 diarrhea, mild oral irritation, or peripheral edema. For patients treated with HDC-ASCT, CTCAE grade-3 toxicities included febrile neutropenia in seven patients and sepsis in two patients. All patients recovered following additional treatments for these side effects.

### Relapses and cause of death

Twenty-three patients presented with relapse of lymphoma, ten after CR following WBRT and 13 after CR following chemotherapy alone. Twelve of 23 patients (52.2 %) relapsed within 2 years following initial diagnosis of PCNSL. At the first relapse, seven patients underwent HD-MTX rechallenge, other five underwent different chemotherapy regimens, five underwent WBRT, and six went to best supportive care. Three patients developed extra-CNS lymphoma, presenting after long follow-up. One patient had lymphoma presenting in the skin at 37 months, one patient developed intestinal lymphoma at 54 months, and another patient developed testicular lymphoma at 97 months.

Thirty-nine (63.9 %) patients died during the study. The cause of death was determined in 38 of 39 patients. Thirty patients out of 39 (76.9 %) died as a result of lymphoma. Four patients died from infection associated with moderate to severe neurological deficits after treatment. Two patients died of intracerebral hemorrhage 4 and 90 months after CR following WBRT. Two patients died of causes unrelated to their lymphoma: one of hepatocellular carcinoma after HD-MTX combined with WBRT, and another of anorexia nervosa after HD-MTX consolidation therapy. One patient suffering from lung cancer was cured, and another survived prostate cancer; both patients had received treatment for PCNSL with HDC-ASCT.

### Survivors and performance scores

The 1-year patient survival rate was 75.4 %, and the 2-year survival rate was 60.7 % for all patients. Of the total patients studied, 36.1 % (22/61) survived with a median follow-up of 59.7 months. The patient survivor group included 70 % (7/10) of the patients who received HDC-ASCT; 56.2 % (9/16) of patients treated with HD-MTX consolidation and 23.1 % (6/23) of patients treated with HD-MTX combined with WBRT (Fig. 4c).

Of the 22 survivors of the study, the median Karnofsky performance status (KPS) was 70 %, with a KPS of 80–100 % in 45.5 % (10/22) of patients (Fig. 4d). The younger the patients were, the better the KPS. Six of the seven survivors (71.4 %) who received HDC-ASCT had returned to work and had a KPS of between 90–100 %.

## Discussion

The median OS of the 61 patients who were included in this study was 41.6 months. The OS of patients in this study is supported by the results of other published series using HD-MTX with or without WBRT for unselected patients with PCNSL where median OS values have been reported as between 8 and 42 months (Hodson et al. 2005; Silvani et al. 2007; Kiewe et al. 2008; Muirhead et al. 2013; Hart et al. 2014). In our intent-to-treat patient population, the patients in their seventh decade had a median OS of 67.6 months, and patients in their eighth decade had the shortest median OS of 14.4 months. Multivariate analysis supported significant effects of age under 65 years and CR by initial HD-MTX therapy on survival time. Those two factors had been applied in selection steps of treatments for our patients consistently.

In this study, patients who responded to chemotherapy had a longer survival time than those who did not (Table 2). Those chemo-sensitive patients might receive benefits from HD-MTX alone or upfront HDC-ASCT strategy. Two different treatments were selected for patients who achieved CR according to age below or above 65 years at Selection Step 3: HDC-ASCT and HD-MTX consolidation therapy. Patients who received HDC-ASCT had the longest survival in our case series, with a 2-year and 5-year survival rate of 100 and 80 % respectively. With chemotherapy alone, patients with a median age of 72 years received consolidation HD-MTX monotherapy and had a median OS of 67.6 months.

Previously published results of studies using upfront HDC-ASCT treatment in PCNSL have been encouraging and also support our findings (Illerhaus et al. 2008; Alimohamed et al. 2012; Kiefer et al. 2012; Schorb et al. 2013). In our study, upfront HDC-ASCT resulted in 5-year survival rates that compared well with previously reported 5-year survival rates of between 44 and 87 % (Illerhaus et al. 2008; Alimohamed et al. 2012; Schorb et al. 2013). Further, in our study there was no observed mortality related to treatment toxicity with HDC-ASCT. Although HDC-ASCT is the standard treatment for a chemo-sensitive systemic relapse in DLBCL without CNSL involvement, the role of HDC-ASCT for treatment of relapsed or refractory PCNSL remains unclear (Abrey et al. 2003). However, because of the risk of toxicity associated with HDC-ASCT, this treatment may be indicated to younger patients without co-morbidity and administered in specialized centers with experience in using ASCT as therapy.

In this study, we selected patients 65 years or older for HD-MTX consolidation treatment after achieving a CR in Selection Step 3. Cobert and colleagues have reported an excellent clinical outcome following HD-MTX monotherapy in patients with PCNSL who had a median age of 62 years (range 23–86 years) (Cobert et al. 2010). These investigators reported treatment regimes of 8 g/m^2^ MTX on an average of 11 cycles, which resulted in a median OS of 84 months (Cobert et al. 2010). In our study, patients treated with HD-MTX as part of their consolidation therapy were older than patients in this previous report, received a lower dose and fewer cycles of MTX, and still had a comparable median OS of 67.6 months. However, the optimal treatment dose and timing of MTX injections in the treatment of PCNSL are still unknown.

Patients over the age of 65 years can be candidates for HD-MTX therapy with proper oncological care from an experienced medical team after a thorough evaluation of their physical condition. Ghesquieres and colleagues reported that age, clinical performance status, and tumor site lost their prognostic significance after 6 months of treatment in their series of 91 patients with PCNSL (Ghesquieres et al. 2013). Our results in older patients with PCNSL support the conclusions of these previous studies, that older patients with poor clinical performance status and brain involvement by lymphoma are at risk of early death, but a favorable long-term outcome is still possible following treatment.

In the 10 years from 2003, the intention at our clinical center had been to use a consistent treatment regime for patients diagnosed with PCNSL. Initial HD-MTX monotherapy was followed by a response-adapted chemotherapy-alone strategy of HDC-ASCT or HD-MTX consolidation deferring WBRT. The treatment decision for each patient was conducted using a three-step selection process. A favorable outcome was seen in some patients treated with chemotherapy alone, which supports our use of a step-wise patient treatment selection process that increases survival of patients with chemo-sensitive PCNSL. In a few previously reported single center studies, the patients who were eligible for treatment with HD-MTX showed a median OS of between 40 and 54 months with or without radiotherapy (Cobert et al. 2010; Makino et al. 2014). An early CR at the second cycle of intensive chemotherapy was a prognostic factor in a previously reported study (Pels et al. 2010). Completion of at least three cycles of HD-MTX-based chemotherapy was of prognostic significance in another study (Makino et al. 2014). These reports indicate that chemo-sensitivity is an important prognostic factor for patients with PCNSL. Our study supports the view that chemo-sensitivity can be assessed during but not before chemotherapy. Markers for chemo-sensitivity are required before commencing HD-MTX or intense chemotherapy, because of treatment-associated morbidity and mortality.

Relapse of lymphoma, both early and delayed, remains a major problem in the treatment of PCNSL, including in this study. The main cause of death of our patients with PCNSL was relapse or progression of lymphoma. In this series, 52.2 % of cases of relapse occurred within 2 years, and late relapse occurred during follow-up after 8 years at most. In the literature, the majority of the recurrences in PCNSL are reported to develop within 2 years after first-line treatment (Jahnke et al. 2006; Nayak et al. 2011). These results support the view that intensive clinical follow-up should continue for at least 2 years following treatment. Treatment toxicity should also be assessed at both early and late follow-up, especially neurotoxicity and secondary neoplasms.

We had treated elderly patients with PCNSL with our chemotherapy-alone strategy for 10 years with acceptable morbidity. Pre-treatment morbidity has previously precluded elderly patients from commencing potentially curative treatment. Attempts to reduce toxicity without compromising efficacy must address three immediate issues: reliable markers for chemo-sensitivity to initial HD-MTX, the optimum regimen to increase ratio of CR following initial chemotherapy, and the optimum consolidation regimen in patients who achieved CR following HD-MTX. Multicenter randomized controlled clinical trial data is awaited to address these questions.

The present study has limitations that are inherent in a retrospective clinical analysis and in this rare and aggressive disease. These limitations include a relatively small study population. A detailed and consecutive evaluation of cognitive function was not included. All patients were sequentially incorporated into the study, and no control patients were available.

## Conclusions

In conclusion, this single-center retrospective clinical study has shown that treatment of PCNSL with upfront HDC-ASCT and consolidation phase HD-MTX monotherapy may be feasible, even for elderly patients in a routine clinical setting, using the three-step selection by eligibility, response to HD-MTX, and age threshold of 65 years for ASCT. Long-term follow-up of patients treated for PCNSL is necessary to assess relapse of lymphoma, or development of neurotoxicity and other malignancy.
